# Clark La Motte Goddard, A.B., D.D.S., A.M.

**Published:** 1905-08

**Authors:** 


### Obituary.

### CLARK LA MOTTE GODDARD, A.B., D.D.S., A.M.

Clark La Motte Goddard, A.B., D.D.S., A.M., died in San Francisco, March 30, 1905, aged fifty-five years.

"Dr. Goddard was born of American parents, in Beloit, Wis., June 26, 1849, where he spent most of his boyhood days, and he also received his early educational training in the public schools of his native city.

"In 1868 he entered the Beloit College, from which he graduated four years later, receiving the degree of Bachelor of Arts, and in 1875 the degree of Master of Arts was conferred upon him by the same institution.

"At an early age he determined to make dentistry his life's work and began the study of it at first with E. M. Clark, M.D., of Beloit, later matriculating at the Philadelphia College of Dentistry and received therefrom his degree of Doctor of Dental Surgery in 1874.

"He first began practice in Chicago, but soon made up his mind to locate in San Francisco, arriving here in 1875, where he has been in practice ever since, associating himself first with Dr. J. L. Cogswell, and in 1876 with Dr. H. E. Knox for four years.

"He was constantly active in all associational work, which is shown by the fact that he became a member of the San Francisco Dental Association and the California State Dental Association in 1875, the California State Odontological Society in 1884, and the American Dental Association in 1890. He was initiated into the Delta Sigma Delta Fraternity in 1892; was made an honorary member of the Washington, D. C., Dental Association in 1895, and of the Oregon State Dental Association in 1897; in 1891 he was the president of the San Francisco Dental Association; a year later he was chairman of the Dental Section of the American Medical Association, and in 1898 was president of the Pacific Coast Dental Congress held in Portland, Oregon.

"In addition to the foregoing he has repeatedly been a delegate to the National Association of Dental Faculties, and has been further honored by election and appointment to various other offices in different societies.

"At the time of organizing the Dental Department of the University of California, in 1882, he was appointed by the regents of the University a member of the faculty, giving practically a continuous service since that time, and the longest in the history of the College.

"The various appointments in that direction were successively, Professor of Mechanical Dentistry; Professor of Mechanical Dentistry, Dental Metallurgy, and Orthodontia; Professor of Orthodontia and Dental Metallurgy; Professor of Orthodontia.

"In 1902 he retired from active membership in the faculty, when he was appointed to the honorary position of Emeritus Professor of Orthodontia, but since then he has given a yearly special course in comparative Odontology. During his long experience in teaching he served as dean of the faculty for eight years at different periods.

"He has contributed largely to dental literature, and has presented valuable treatises, some of which have been included in recent text-books and accepted as the best authority on the subjects selected. Among them might be mentioned the chapters in the 'American Text-Book of Prosthetic Dentistry' on the 'Principles of Metal Work and Orthodontia Technic,' and on 'Cast

Dentures of Aluminum and Fusible Alloys;' and in the 'American Text-Book of Operative Dentistry' the chapter on the 'Management of Deciduous Teeth' and another on 'Orthodontia.'

"Dr. Goddard married Miss Emily Louise Bunker, August 13, 1881, and as an issue of this union there are two children, a son and a daughter, all of whom survive him."

We are indebted for the foregoing facts in the life of Dr. Goddard to the *Pacific Dental Gazette.* The announcement of the death of this eminent practitioner came as a serious blow to his many friends in the East. He was probably better known personally than most of our coworkers on the Pacific coast. His valuable contributions to dental literature made his name familiar to those who were not brought in personal contact with him at the various conventions in which he was so frequently an active participant. At the meetings of the Association of Faculties his presence and cultured manner in debate held his audience in many of the stormy sessions of that body and largely aided in producing conviction for the side he advocated. Whether in opposition or in favor of any disputed question, his message always commanded respect.

His death at a comparatively early age is a serious loss, not to the Pacific coast only, but to the larger body throughout the country, for he labored earnestly for the broader professional life based upon a true professional spirit, unfortunately too much lacking in dental circles in this country. His work and example should be a stimulus to higher ideals and more important results.

